# Hypoxia Inducible Factor-1α in Astrocytes and/or Myeloid Cells Is Not Required for the Development of Autoimmune Demyelinating Disease^1^,^2^,^3^

**DOI:** 10.1523/ENEURO.0050-14.2015

**Published:** 2015-04-20

**Authors:** Natacha Le Moan, Kim M. Baeten, Victoria A. Rafalski, Jae Kyu Ryu, Pamela E. Rios Coronado, Catherine Bedard, Catriona Syme, Dimitrios Davalos, Katerina Akassoglou

**Affiliations:** 1 Gladstone Institute of Neurological Disease, University of California, San Francisco, San Francisco, CA 94158, USA; 2 Department of Neurology, University of California, San Francisco, San Francisco, CA 94143, USA

**Keywords:** astrocytes, cre/loxP, EAE, HIF-1alpha, macrophages, neuroinflammation

## Abstract

Hypoxia-like tissue alterations, characterized by the upregulation of hypoxia-inducible factor-1α (HIF-1α), have been described in the normal appearing white matter and pre-demyelinating lesions of multiple sclerosis (MS) patients. As HIF-1α regulates the transcription of a wide set of genes involved in neuroprotection and neuroinflammation, HIF-1α expression may contribute to the pathogenesis of inflammatory demyelination. To test this hypothesis, we analyzed the effect of cell-specific genetic ablation or overexpression of HIF-1α on the onset and progression of experimental autoimmune encephalomyelitis (EAE), a mouse model for MS. HIF-1α was mainly expressed in astrocytes and microglia/macrophages in the mouse spinal cord at the peak of EAE. However, genetic ablation of HIF-1α in astrocytes and/or myeloid cells did not ameliorate clinical symptoms. Furthermore, conditional knock-out of Von Hippel Lindau, a negative regulator of HIF-1α stabilization, failed to exacerbate the clinical course of EAE. In accordance with clinical symptoms, genetic ablation or overexpression of HIF-1α did not change the extent of spinal cord inflammation and demyelination. Overall, our data indicate that despite dramatic upregulation of HIF-1α in astrocytes and myeloid cells in EAE, HIF-1α expression in these two cell types is not required for the development of inflammatory demyelination. Despite numerous reports indicating HIF-1α expression in glia, neurons, and inflammatory cells in the CNS of MS patients, the cell-specific contribution of HIF-1α to disease pathogenesis remains unclear. Here we show that although HIF-1α is dramatically upregulated in astrocytes and myeloid cells in EAE, cell-specific depletion of HIF-1α in these two cell types surprisingly does not affect the development of neuroinflammatory disease. Together with two recently published studies showing a role for oligodendrocyte-specific HIF-1α in myelination and T-cell-specific HIF-1α in EAE, our results demonstrate a tightly regulated cellular specificity for HIF-1α contribution in nervous system pathogenesis.

## Abstract (precis)

Despite numerous reports indicating HIF-1α expression in glia, neurons, and inflammatory cells in the CNS of MS patients, the cell-specific contribution of HIF-1α to disease pathogenesis remains unclear. Here we show that although HIF-1α is dramatically upregulated in astrocytes and myeloid cells in EAE, cell-specific depletion of HIF-1α in these two cell types surprisingly does not affect the development of neuroinflammatory disease.

## Significance Statement

Despite numerous reports indicating HIF-1α expression in glia, neurons, and inflammatory cells in the CNS of MS patients, the cell-specific contribution of HIF-1α to disease pathogenesis remains unclear. Here we show that although HIF-1α is dramatically upregulated in astrocytes and myeloid cells in EAE, cell-specific depletion of HIF-1α in these two cell types surprisingly does not affect the development of neuroinflammatory disease. Together with two recently published studies showing a role for oligodendrocyte-specific HIF-1α in myelination and T-cell specific HIF-1α in EAE, our results demonstrate a tightly regulated cellular specificity for HIF-1α contribution in nervous system pathogenesis.

## Introduction

Multiple sclerosis (MS) is a chronic inflammatory demyelinating disease of the nervous system characterized by inflammation, gliosis, destruction of myelin sheaths, and axonal damage leading to permanent functional deficits (Brück and Stadelmann, 2005). Hypoxia-like tissue alterations, predominantly characterized by the accumulation of the hypoxia-inducible factor-1α (HIF-1α), occur at a very early stage in MS pathogenesis (Aboul-Enein et al., 2003; Graumann et al., 2003; Lassmann, 2003; Stadelmann et al., 2005; Marik et al., 2007; Zeis et al., 2008). Indeed, HIF-1α is upregulated in pre-demyelinating lesions and normal appearing white matter (NAWM) of MS patients (Graumann et al., 2003; Zeis et al., 2008). Whereas HIF-1α expression is usually minimal or absent from active white matter lesions (Aboul-Enein et al., 2003; Marik et al., 2007), it is highly expressed in oligodendrocytes specifically in lesions with distal dying-back oligodendrogliopathy (Lassmann, 2003). Early hypoxic changes occurring within pre-demyelinating plaques are associated with microglia activation, perivascular accumulation of lymphocytes, blood–brain barrier (BBB) disruption and mild axonal injury (Marik et al., 2007). Determining the early hypoxic events leading to the formation of demyelinating lesions and their causative role in the pathology is crucial to better understand MS pathogenesis and discover novel targets and strategies for therapeutic intervention.

In the present study, we examined whether HIF-1α expression regulates the onset and progression of inflammatory demyelination. HIF-1α is a heterodimeric transcription factor that orchestrates the genetic response to hypoxic and inflammatory conditions by activating the transcription of a wide set of genes that regulate several biological processes, including cellular proliferation, survival, angiogenesis, and glucose and iron metabolism (Sharp and Bernaudin, 2004; Majmundar et al., 2010). A central regulator of HIF-1α turnover is Von Hippel Lindau (VHL), an ubiquitin ligase targeting HIF-1α for degradation (Kaelin, 2008). HIF-1α function in the nervous system can be either protective by increasing the survival of neurons and oligodendrocytes (Baranova et al., 2007; Vangeison et al., 2008), or damaging by inducing BBB disruption (Argaw et al., 2006; Weidemann et al., 2009). The role of HIF-1α in inflammation has been examined in animal models of sepsis (Peyssonnaux et al., 2007; Thiel et al., 2007), rheumatoid arthritis and chronic cutaneous inflammation (Cramer et al., 2003) using conditional gene targeting approaches allowing tissue-specific deletion of HIF-1α and VHL. Altogether these studies described that overexpression of HIF-1α results in hyperinflammatory responses and increased vascular permeability (Weidemann et al., 2009), whereas ablation of HIF-1α decreases inflammation (Cramer et al., 2003; Peyssonnaux et al., 2007; Thiel et al., 2007). Although the role of HIF-1α in peripheral inflammatory responses is well defined, whether glial-cell-specific expression of HIF-1α plays a protective or damaging effect in inflammatory demyelination is unknown.

Here we show that similar to MS lesions, HIF-1α is induced mainly in astrocytes and microglia/macrophages in white matter areas in experimental autoimmune encephalomyelitis (EAE), a mouse model for MS. Surprisingly, cell-specific genetic ablation of HIF-1α in astrocytes and/or myeloid cells does not change disease onset or progression. Overall, we find that despite considerable upregulation of HIF-1α in glia and monocytes during neuroinflammation, astrocyte-specific and/or myeloid-cell-specific expression of HIF-1α does not affect the development of inflammatory demyelinating disease.

## Material and Methods

*Mice*. Six-week-old female C57BL/6 mice were purchased from Charles River Laboratories. *HIF-1α^fl/fl^* C57BL/6 (Ryan et al., 2000), *VHL^fl/fl^* C;129S (Haase et al., 2001), and *HIF-1α^luc^* FVB/NJ (Safran et al., 2006) mice were obtained from The Jackson Laboratory. *VHL^fl/fl^* C;129S and *HIF-1α^luc^* FVB/NJ mice were backcrossed at least six generations with pure C57BL/6 mice to perform EAE experiments. We then crossed *HIF-1α^fl/fl^* mice with mice expressing the cre recombinase driven by the lysozyme M promoter (*lysM-Cre*; Clausen et al., 1999) or the glial fibrillary acidic protein (GFAP) promoter (*GFAP-Cre*; Bajenaru et al., 2002) to generate cell-specific depletion of HIF-1α in microglia/macrophages or astrocytes respectively. *lysM-Cre* mice were also crossed with *VHL^fl/fl^* to generate cell-specific overexpression of HIF-1α in the myeloid lineage. Mice were genotyped using the following primers: *HIF-1α^luc^*: forward, 5'-CGGTATCGTAGA GTCGAGGCC-3'; reverse, 5'-GGTAGTGGTGGCATTAGCAGTAG-3' to detect the ODD-Luc cDNA and forward, 5'-AAGGGAGCTGCAGTGGAGTA-3'; reverse, 5'-CCGAAAATC TGTGGGAAGTC-3' to detect the WT cDNA locus; *HIF-1α^fl/fl^*: forward, 5'-GTTGGGGCA GTACTGGAAAG-3'; reverse, 5'-TGCTCATCAGTTGCCACTT-3'; *VHL^fl/fl^*: forward, 5'-CAGCTTGCGAATCCGAGGGAC-3'; reverse, 5'-CCTTCTGTCTTGGCCTCCTGAG-3'; *GFAP-Cre*: forward, 5'-ACTCCTTCATAAAGCCCTCGCATCCC-3'; reverse, 5'-ATCACT CGTTGCATCGACCG-3'; *lysozyme M-Cre*: forward, 5'-CTTGGGCTGCCAGAATTTCTC-3'; reverse, 5'-CCCAGAAATGCCAGATTACG-3' and reverse, 5'-TTACAGTCGGCCAGGCTG AC-3' as internal control. All animal procedures are performed according to the regulation of University of California, San Francisco's animal care committee.

*EAE induction and clinical assessment*. EAE was induced in 6-wk-old female mice in the C57BL/6 background by subcutaneous injection of 50 µg MOG_35–55_ (myelin oligodendrocyte glycoprotein; CPC Scientific) in complete Freund's adjuvant (CFA) (Sigma-Aldrich) supplemented with 200 ng of heat-inactivated mycobacterium tuberculosis H37Ra (Difco Laboratories) as previously described (Adams et al., 2007). Control mice were injected only with CFA, as indicated. Mice were injected intravenously with 200 ng pertussis toxin (PTX; Sigma-Aldrich) on days 0 and 2 of the immunization. Mice were scored daily as follows: 0, no symptoms; 1, loss of tail tone; 2, ataxia; 3, hindlimb paralysis; 4, hindlimb and forelimb paralysis; and 5, moribund.

Ex vivo *luciferase assay*. Proteins from mouse spinal cords were extracted on ice with a homogenizer in the Passive Lysis Buffer (Promega) supplemented with Protease and Phosphatase inhibitor cocktail set (Calbiochem). After a centrifugation at 13,000 × *g* for 20 min at 4 °C, 20 μL of the resulting supernatant was mixed with 50 μL of luciferase substrate (Luciferin, Promega) and the luminescence was measured on the Monolight 2010 luminometer (BD Biosciences). Relative light units (RLUs) for luciferase were normalized to the optical density (OD) at 600 nm of respective spinal cord lysates.

*Induction of hypoxia*. Hypoxia was induced as previously described (Le Moan et al., 2011). Briefly, adult WT mice were exposed to 8% O_2_ for 6 h in an O_2_ chamber controlled by the ProOx P110 and ProCO_2_ P120 systems (BioSpherix). Control mice were kept in the same room under ambient O_2_. Mice were killed and brains lysed on ice in lysis buffer (100 mm NaCl, 1 mm EDTA, and 20 mm Tris-HCl, pH 7.4, 10% glycerol) containing 0.5% Tween 20 and supplemented with protease and phosphatase inhibitor cocktail (Calbiochem). Protein extracts were then analyzed for HIF-1α levels by Western blot.

*Western blotting*. Proteins from mouse tissues were extracted on ice with a homogenizer in a lysis buffer containing the following: 50 mm Tris-HCl, pH 7.5, 150 mm NaCl, 1% NP-40, 5 mm EGTA, 5 mm EDTA, and 20 mm NaF, and supplemented with Protease and Phosphatase inhibitor cocktail set (Calbiochem). The tissue lysates were cleared by a centrifugation at 13,000 × *g* for 20 min at 4 °C. Protein concentration of the resulting supernatant was determined by the Bio-Rad protein assay (Bio-Rad). Equal amounts of tissue extracts (60 μg) dissolved in Laemmli Buffer were separated by 8–16% SDS-PAGE and Western blotting was performed as previously described (Le Moan et al., 2011). The membranes were probed with the rabbit polyclonal anti-mouse HIF-1α antibody (1:500, Novus Biological) and the mouse monoclonal anti-β-actin antibody (1:10,000, Sigma-Aldrich) and developed by chemiluminescence (ECL Plus, GE Healthcare).

*Quantitative real-time PCR.* Total RNA was extracted from spinal cords using the RNeasy kit (Qiagen) according to the manufacturer instructions. Reverse transcription and real-time PCR (RT-PCR) on a StepOnePlus Real-Time PCR System (Applied Biosystems) were performed as previously described (Le Moan et al., 2011) in a 25 μL reaction using 2 μL of cDNA template, 12.5 μL of SYBR Green PCR Master Mix (Applied Biosystems), and 1 μL of the following sense and antisense primers: inducible nitric oxide synthase (*iNOS*): forward, 5'-GGCAAACCCAAGGTCTACGTT-3'; reverse, 5'-TCGCTCAAG TTCAGCTTGGT-3'; erythropoietin (*EPO*): forward, 5'-CATCTGCGACAGTCGAGTTCTG-3'; reverse, 5'-CACAACCCATCGTGACATTTT C-3', *HIF-1α*: forward, 5'-ACCTTCATCGGAAACTCC AAA G-3'; reverse, 5'-CTGTTAGGCTGGGAAAAGTTA GG-3'; *L7*: forward, 5'-GAAGCT CATCTATGAGAAGGC-3'; reverse, 5'-AAGACGAAGGAGCTGCAGAAC-3'. Thermocycling conditions were the following: initial step of 10 min at 95ºC, and then 40 cycles of 15 s denaturation at 95 °C and 1 min annealing and extension at 60 °C. Results were analyzed with the StepOne Software v2.0 using the comparative CT method. Transcripts of gene of interest were normalized against the transcripts of the mouse ribosomal protein L7, used as a housekeeping gene, and were presented as fold-change versus the L7 transcript content. Each RT-PCR was performed in triplicate and repeated in three different animals.

*Immunohistochemistry.* Mice were anesthetized and transcardially perfused with ice-cold PBS solution containing zinc (3.9 mg/ml) as previously described (Sun et al., 2008). Brains and spinal cords were dissected and frozen in OCT-compound. Sections of 10 µm thickness were fixed overnight in a zinc fixative solution (0.1 m Tris, pH 7.4, 0.05% calcium acetate, 0.5% zinc acetate, and 0.5% zinc chloride) and immunostained overnight at 4 °C with a rabbit polyclonal anti-mouse HIF-1α antibody (1/500, Novus Biological). For light microscopy immunostaining, HIF-1α signal was developed by the peroxidase substrate 3-amino-9-ethylcarbazole (Sigma-Aldrich). The signal specificity was determined by omission of the primary antibody and by pre-adsorption of the primary antibody with the NB100-449 blocking peptide (Novus Biological). Double-immunofluorescence staining was performed with antibodies against GFAP (rat anti-GFAP: 1:1000, Zymed Laboratories) and Isolectin B4 (biotin IsoB4: 1/300, Sigma-Aldrich). Images were acquired with an Axioplan II epifluorescence microscope (Carl Zeiss) equipped with dry Plan-Neofluar objectives (10× 0.3 NA, 20× 0.5 NA, or 40× 0.75 NA) using an Axiocam HRc CCD camera and the Axiovision image analysis software.

*Histopathology*. Histopathologic analysis and quantitation of inflammation and demyelination in mouse tissue were performed on paraffin sections as previously described (Adams et al., 2007). Sections were stained with hematoxylin/eosin and luxol fast blue/periodic acid-Schiff stain (LFB/PAS). Demyelinated areas were quantified on LFB/PAS stained sections using Axiovision software. Areas of demyelination were manually outlined by an observer blinded to the genotype, and measured using Axiovision software. For each mouse, the sum of the demyelinated areas was divided by the number of coronal sections throughout the cord that were quantified (on average 8 per cord). Inflammation was assessed by immunohistochemistry following antigen retrieval in boiled target retrieval solution (Dako) using an antibody against CD3 (rabbit anti-CD3, 1/500; Abcam) and a secondary anti rabbit-cy3 (1/300, JAX). Anti-CD3+ cells were counted on coronal sections throughout the cord (typically 8 per mouse) within regions of interest. Regions-of-interest consisted of 20 boxes of 100 µm × 100 µm per cross section, placed in areas of parenchymal and meningeal inflammation. To detect macrophage infiltration we used rat anti-mouse Mac-2 (1:200, CEDARLANE) followed by a secondary anti rat-FITC (1:300, JAX). Images were acquired by a Keyence Biorevo BZ-9000E and measurement of Mac-2+ area was performed using ImageJ. To detect iNOS-expressing astrocytes, we performed double-immunofluorescence staining with antibodies against iNOS (1:100, Abcam) and GFAP (1:1000, Zymed Laboratories). Images were acquired with an Axioplan II epifluorescence microscope (Carl Zeiss) and labeled cells were counted in the central area of spinal cord coronal sections within a region of interest (box of 200 × 280 µm, six different rostrocaudal sections per animal). Quantification was performed by observers blinded to the genotypes of the mice.

*Statistical analyses*. Differences between experimental conditions were analyzed with ANOVAs and *t* tests, and *p* values were corrected for multiple comparisons using the method of Holm or Tukey’s Honest Significant Difference. When normality and homoscedasticity assumptions were violated, data were transformed using a natural logarithmic transformation. For daily scoring of clinical score data, we fit a linear mixed-effects model (Laird and Ware, 1982; Lee et al., 2003) using the lme4 package (Bates et al., 2013) in R (R Core Team, 2013). We used the fitted model to obtain estimates of the mean day of onset and the mean of the maximum clinical score for each genotype group. We performed a Fisher’s exact test to determine whether there is a significant relationship between genotype and mice that achieve score 3 or higher during the experiment (percentage paralysis). Power and sample size analyses were performed with SAS 9.3. Data are shown as mean ± SEM; *p* < 0.05 was considered significant. Single comparisons to control were made using an unpaired *t* test.

## Results

### HIF-1α stabilization is increased in the mouse spinal cord at the peak of EAE

**Figure 1:**
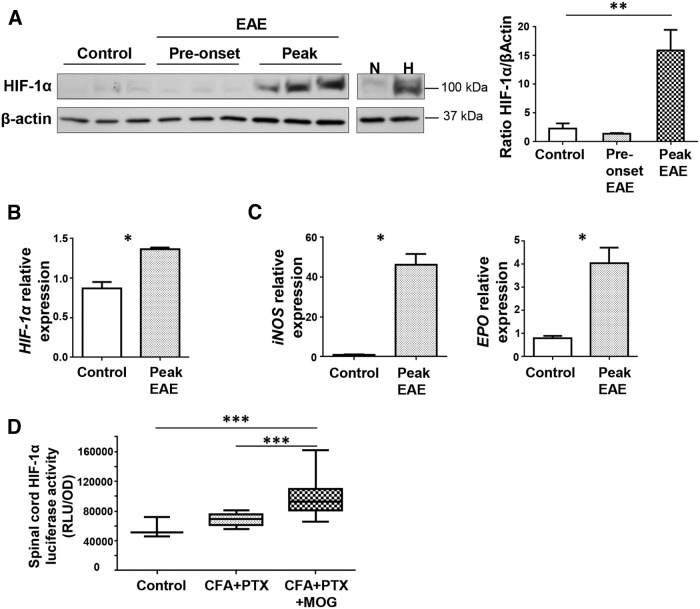
Stabilization and transcriptional activation of HIF-1α in the spinal cord of EAE mice. ***A***, Western blot for HIF-1α in spinal cords of control (*n* = 3), pre-onset EAE, day 7 postimmunization (p.i.; *n* = 3) and peak EAE, day 16 p.i. (*n* = 3) mice. Normoxic (21% O_2_) and hypoxic (10% O_2_) brain extracts were loaded with spinal cord extracts of EAE mice to demonstrate HIF-1α protein stabilization. β-Actin-loading controls were performed on the same membrane. N, Normoxia; H, hypoxia. ANOVA: *p* = 0.002*^a^*. Peak EAE had a higher ratio of HIF-1α/β-actin than control (*p* = 0.006*^a^*) or pre-onset EAE mice (*p* = 0.003*^a^*). ***B***, Quantitative real-time PCR analysis of *HIF-1α* expression in spinal cords of control (*n* = 3) and peak EAE (*n* = 3) mice, *p* = 0.01*^b^*. ***C***, Quantitative real-time PCR analysis of HIF-1α target genes *iNOS* and *EPO* in spinal cords of control (*n* = 3) and peak EAE (*n* = 3) mice, *p* = 0.03*^c^* and *p* = 0.04*^d^*. ***D***, Quantitative analysis of photon emission in spinal cord of *HIF-1α^luc^* control (*n* = 3) and peak EAE mice with MOG/CFA/PTX (*n* = 12) or CFA/PTX (*n* = 15). ANOVA: *p* = 0.00004*^e^*. MOG/CFA/PTX had greater bioluminescence than both CFA/PTX (*p* = 0.0005*^e^*) and control mice (*p* = 0.0005*^e^*). *HIF-1α^luc^* mice were killed and spinal cord tissues were excised to extract proteins. The bioluminescence signal was quantified *ex vivo* by the luciferase assay and expressed as RLUs per OD of proteins at 600 nm. Data are presented as mean ± SEM; *p < 0.05, **p < 0.01, ***p < 0.001 by Holm’s test in ***B*** and ***C***, and one-way ANOVA followed by Tukey multiple comparisons in ***A*** and ***D*** . Superscript letters refer to the statistical results in Tables 1, 2, and Results.

To determine the temporal regulation of HIF-1α in neuroinflammatory disease, we compared HIF-1α expression in spinal cord extracts from EAE mice at the onset (day 7) and peak of disease (day 16) with healthy control mice (nonimmunized; Fig. 1A). HIF-1α protein levels at the peak of EAE were ∼7-fold higher compared with control (Fig. 1A). HIF-1α was not detected in control mice or at the onset of EAE (Fig. 1A). Protein extracts from normoxic and hypoxic brains were used as a positive control of HIF-1α accumulation (Fig. 1A). *HIF-1α* RNA levels were ∼1.5-fold higher at the peak of EAE (Fig. 1B), suggesting that HIF-1α expression is mainly regulated at the protein rather than the transcriptional level. In accordance, RNA expression analysis of HIF-1α target genes *iNOS* and *EPO* showed significant increase at the peak of EAE (Fig. 1C). Furthermore, we induced EAE in HIF-1α genetic reporter mice (*HIF-1α^luc^*), which express the luciferase gene fused to the oxygen-sensing domain of HIF-1α and used *ex vivo* bioluminescence assay as a sensitive and accurate strategy to quantify HIF-1α accumulation in spinal cord extracts. Consistent with our biochemical results, the luciferase signal was significantly increased in MOG_35–55_-immunized mice at the EAE peak compared with control (Fig. 1D). Because exposure to bacterial pathogens, such as PTX, may increase HIF-1α accumulation (Zinkernagel et al., 2007), we analyzed whether HIF-1α is upregulated in the absence of the MOG_35–55_ peptide. Immunization of *HIF-1α^luc^* mice with only CFA and PTX did not increase luciferase signal (Fig. 1D), indicating that HIF-1α is specifically upregulated in response to MOG_35–55_-induced EAE.

### HIF-1α is expressed in astrocytes and microglia/macrophages in the mouse spinal cord at the peak of EAE

**Figure 2:**
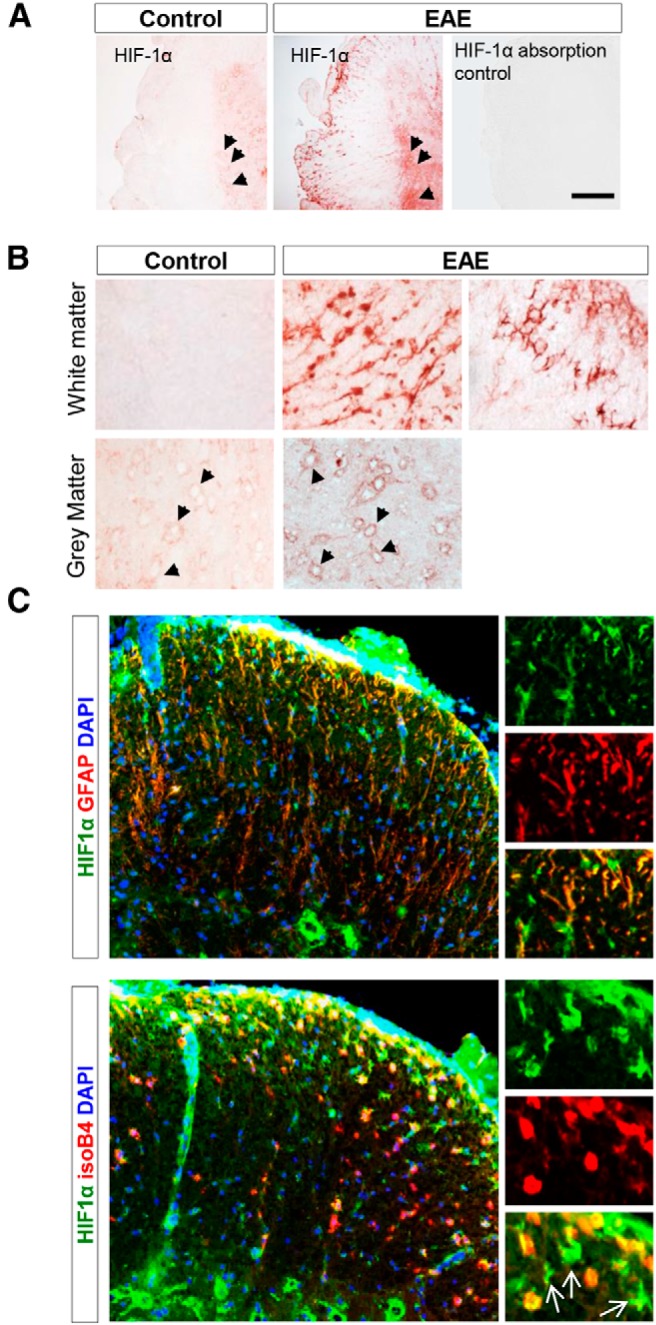
HIF-1α is expressed in astrocytes and microglia/macrophages in the spinal cord of EAE mice. ***A***, Immunohistochemical staining for HIF-1α in spinal cord sections from healthy mice and at peak EAE, 17 d p.i. Absorption control after anti-HIF-1α antibody incubation with HIF-1α blocking peptide at peak EAE. Scale bar, 300 μm. Arrows indicate neuronal cells. ***B***, Higher-magnification of HIF-1α immunostaining in the grey and white matter of the spinal cord of healthy and EAE mice. Scale bar, 50 μm. Arrows indicate neuronal cells. ***C***, Double-immunofluorescence with antibodies against HIF-1α (green) and GFAP (top) or isoB4 (bottom) (red) of spinal cord sections of EAE mice 17 d after immunization. Scale bar, 50 μm. Arrows indicate HIF-1α^+^isoB4^–^ cells with astrocyte-like morphology (green).

In MS lesions, HIF-1α is mainly upregulated in astrocytes and oligodendrocytes in the NAWM (Graumann et al., 2003; Zeis et al., 2008), neurons, astrocytes, and oligodendrocytes in pre-demyelinating MS lesions (Aboul-Enein et al., 2003; Marik et al., 2007), and in oligodendrocytes in active lesions with dying-back oligodendrogliopathy (Lassmann, 2003). We thus examined the cell-type-specific expression of HIF-1α in spinal cords of EAE mice. Similar to our previous biochemical observations, immunohistochemistry showed HIF-1α accumulation in mouse spinal cords at the peak of EAE (Fig. 2A). As expected, preincubation of the HIF-1α antibody with a blocking peptide abolished the staining (Fig. 2A), indicating that the signal detected is specific for HIF-1α. HIF-1α staining showed a homogenous and widespread pattern of expression both in grey and white matter areas of spinal cord tissue of EAE mice (Fig. 2A). HIF-1α was weakly expressed in neurons of the grey matter in healthy control mice (Fig. 2A). At EAE peak, HIF-1α was increased in neurons of the grey matter and induced in the white matter (Fig. 2A). Higher-magnification images show prominent staining of HIF-1α in different cell types in the white matter area of the spinal cord of EAE mice (Fig. 2B). To identify the cell types that express HIF-1α in the white matter after EAE, we performed double-immunofluorescence staining of HIF-1α with cell-specific markers. At the peak of EAE, HIF-1α colocalized with the astrocyte marker GFAP and the microglia/macrophage marker IsoB4 (Fig. 2C), indicating that astrocytes and microglia/macrophages are the two major cell types that express HIF-1α in myelinated areas in EAE.

### Genetic ablation or overexpression of HIF-1α in astrocytes or the myeloid lineage does not change the clinical course of EAE or demyelination

To determine whether HIF-1α regulates the onset and progression of inflammatory demyelination, we generated four different mouse strains with cell-specific genetic depletion or overexpression of HIF-1α in astrocytes and myeloid cells. We generated cell-specific depletions of HIF-1α by crossing *HIF-1α^fl/fl^* mice with *lysM-Cre* and/or *GFAP-Cre* mice, which allows specific depletion of HIF-1α in the myeloid lineage and astrocytes, respectively. *lysM-Cre:HIF-1α^fl/fl^* and *GFAP-Cre:HIF-1α^fl/fl^* mice have extensive loss of HIF-1α in targeted cells, have a normal life span, do not display any obvious phenotypes, and show no obvious neurologic defects (Cramer et al., 2003; Weidemann et al., 2009). To determine whether there is a synergistic effect between astrocyte and monocyte expressed HIF-1α, we also generated *GFAP-cre:lysM-Cre:HIF-1α^fl/fl^* double-conditional knock-out mice to achieve simultaneous HIF-1α depletion in both cell types. In addition, we generated mice to examine the role of targeted overexpression of HIF-1α via conditional depletion of VHL. *GFAP-Cre*:*VHL^fl/fl^* mice exhibit severe locomotive defects, hydrocephalus, and increased lethality at 6 weeks (Weidemann et al., 2009) that precludes their analysis in EAE. Therefore, we only generated *lysM-Cre:VHL^fl/fl^* mice.

**Figure 3:**
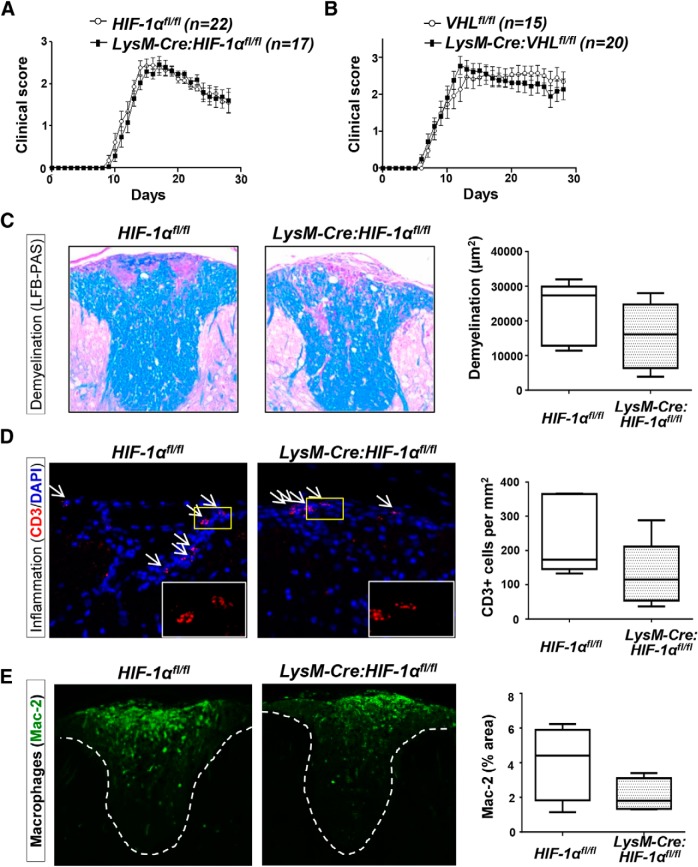
Genetic depletion or overexpression of HIF-1α in myeloid cells does not affect the clinical course of EAE, demyelination, and inflammatory infiltrates. ***A***, Clinical scores of mice genetically depleted for HIF-1α in myeloid cells *lysM-Cre:HIF-1α^fl/fl^* (*n* = 17) and control mice *HIF-1α^fl/fl^* (*n* = 22). ***B***, Clinical scores of mice overexpressing HIF-1α in microglia/macrophages *lysMCre:VHL^fl/fl^* (*n* = 20) and control mice *VHL^fl/fl^* (*n* = 15). ***A*** and ***B***, For each genotype, day of onset, maximum clinical score, and percentage of mice developing signs of paralysis (score ≥3) were calculated using mixed linear effects models, and the results are presented in Table 1. Data are presented as mean ± SEM. ***C***, LFB/PAS staining shows demyelination in *HIF-1α^fl/fl^* (*n* = 5) and *lysM-Cre:HIF-1α^fl/fl^* (*n* = 5). Data are presented as mean ± SEM. Statistical analysis was performed using the unpaired *t* test (*p* = 0.28*^l^*). ***D***, Immunohistochemistry for CD3 shows no significant differences in inflammation between *HIF-1α^fl/fl^* (*n* = 5) and *lysM-Cre:HIF-1α^fl/fl^* (*n* = 5). Data are expressed as number of CD3+ cells/mm^2^ per mouse and presented as mean ± SEM. Statistical analysis was performed using the unpaired *t* test (*p* = 0.14*^m^*). ***E***, Immunohistochemistry for Mac-2 shows no significant differences in inflammation between *HIF-1α^fl/fl^* (*n* = 4) and *lysM-Cre:HIF-1α^fl/fl^* (*n* = 4). Data are expressed as percentage of Mac-2+ area per mouse and presented as mean ± SEM. Statistical analysis was performed using unpaired *t* test (*p* = 0.153*^n^*). Superscript letters refer to the statistical results in Tables 1, 2, and Results.

We induced EAE with the MOG_35–55_ peptide and analyzed clinical signs in the four different mouse strains. Genetic depletion or overexpression of HIF-1α in monocytes did not change the severity of clinical symptoms of EAE (Fig. 3A,B). No differences were observed in day of onset, maximum clinical score, and percentage of paralysis in *lysM-Cre:HIF-1α^fl/fl^*, *lysM-Cre:VHL^fl/fl^*, and *HIF-1α^fl/fl^* mice (Fig. 3A,B; Table 1). Histopathological analysis at EAE peak showed no significant differences in the extent of demyelination, T cell, and macrophage infiltration between *lysM-Cre:HIF-1α^fl/fl^* and *HIF-1α^fl/fl^* littermate control mice (Fig. 3C–E).

**Table 1:** Quantification of day of onset, clinical score, and paralysis in EAE experiments

Experiment	Genotype	Mice, *n*	Day of onset (95% CI)	*p*	Maximum clinical score(95% CI)	*p*	Paralysis, %	*p*
1	*lysM-Cre:HIF-1α^fl/fl^*	17	11.7 (11.0–12.5)	0.77*^f^*	2.71 (2.43–2.98)	0.77*^g^*	23.5	0.73*^h^*
	*HIF-1α^fl/fl^*	22	11.4 (11.0–12.0)		2.79 (2.54–3.02)		31.8	
2	*lysM-Cre:VHL^fl/fl^*	20	7.95 (7.0–9.5)	0.90*^i^*	2.95 (2.32–3.52)	0.90*^j^*	50	0.99*^k^*
	*VHL^fl/fl^*	15	8.56 (7.0–10.0)		2.82 (2.15–3.47)		46.7	
3	*GFAP-Cre:HIF-1α^fl/fl^*	24	11.40 (11.0–12.0)	0.99*^s^*	2.81 (2.43–3.17)	0.99*^t^*	62.5	0.78*^u^*
	*HIF-1α^fl/fl^*	27	11.29 (11.0–12.0)		2.96 (2.64–3.26)		55.6	
4	*GFAP-Cre:lysM-Cre:HIF-1α^fl/fl^*	16	11.44 (10.5–12.0)	0.65*^v^*	2.56 (2.26–2.88)	0.98*^w^*	12.5	0.19*^x^*
	*HIF-1α^fl/fl^*	13	11.98 (11.5–12.5)		2.57 (2.27–2.86)		38.5	

Experiment 1: EAE induction in *lysM-Cre:HIF-1α^fl/fl^* versus control mice *HIF-1α^fl/fl.^* Experiment 2: EAE induction in *lysMCre:VHL^fl/fl^* versus control mice *VHL^fl/fl^*. Experiment 3: EAE induction in *GFAP-Cre:HIF-1α^fl/fl^* versus control mice *HIF-1α^fl/fl^*. Experiment 4: EAE induction in *GFAP-Cre:lysM-Cre:HIF-1α^fl/fl^* versus control mice *HIF-1α^fl/fl^*. Day of onset defined as the first day that the score is ≥0.5. A linear mixed-effects model was used to estimate means and 95% confidence interval (CI) for both day of onset and maximum clinical score for each genotype group. A Fisher’s exact test was used to determine whether there is a significant relationship between genotype and mice that achieve score 3 or higher during the experiment (percentage paralysis). Superscript letters refer to the statistical results in Figures 3A, 3B, 5A and 5B. Results, and Table 2.

**Table 2:** Statistical table

	Data structure	Type of test	Power
*a*	Natural log of HIF1 alpha protein has a normal and homoscedastic distribution and was used as outcome variable	ANOVA followed by Tukey multiple comparisons of means	Peak EAE vs control = 0.99;Peak EAE vs pre-onset EAE = 0.999;Pre-onset EAE vs Control = 0.10
*b*	Normal distribution	Holm’s test	0.911
*c*	Normal distribution	Holm’s test	>0.99
*d*	Normal distribution	Holm’s test	0.89
*e*	Natural log of luciferase activity has a normal and homoscedastic distribution and was used as outcome variable	ANOVA followed by Tukey multiple comparisons of means	CFA+PTX vs control = 0.37;MOG+CFA+PTX vs control = 0.99;MOG+CFA+PTX vs CFA+PTX = 0.99
*f*	Non-normal distribution	Mixed-effects model	0.64
*g*	Non-normal distribution	Mixed-effects model	0.17
*h*	Non-normal distribution	Fisher’s exact test	0.06
*i*	Non-normal distribution	Mixed-effects model	0.46
*j*	Non-normal distribution	Mixed-effects model	0.08
*k*	Non-normal distribution	Fisher’s exact test	0.05
*l*	Normal distribution	Student’s *t* test	0.18
*m*	Normal distribution	Student’s *t* test	0.30
*n*	Normal distribution	Student’s *t* test	0.26
*o*	Normal distribution	Student’s *t* test with Welch’s correction	0.98
*p*	Normal distribution	Student’s *t* test with Holm’s test	0.50
*q*	Normal distribution	Student’s *t* test with Holm’s test	0.67
*r*	Normal distribution	Student’s *t* test with Holm’s test	0.26
*s*	Non-normal distribution	Mixed-effects model	0.08
*t*	Non-normal distribution	Mixed-effects model	0.47
*u*	Non-normal distribution	Fisher’s exact test	0.06
*v*	Non-normal distribution	Mixed-effects model	0.61
*w*	Non-normal distribution	Mixed-effects model	0.03
*x*	Non-normal distribution	Fisher’s exact test	0.22
*y*	Normal distribution	ANOVA	*HIF-1α^fl/fl^* vs *lysM-Cre:HIF-1α^fl/fl^* = 0.03;*HIF-1α^fl/fl^* vs *GFAP-Cre:HIF-1α^fl/fl^* = 0.02;*lysM-Cre:HIF-1α^fl/fl^* vs *GFAP-Cre:HIF-1α^fl/fl^* = 0.03

Superscript letters refer to the statistical tests in figures, Results, and Table 1.

**Figure 4:**
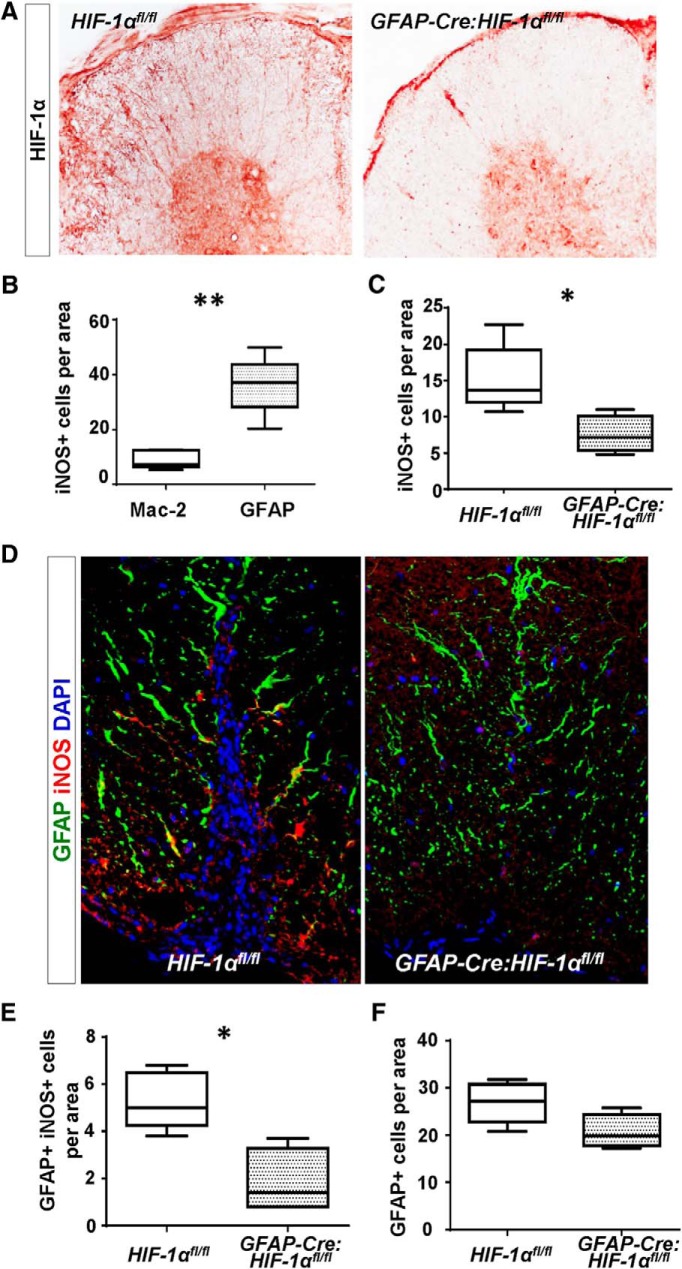
Genetic depletion of HIF-1α in astrocytes decreases expression of HIF-1α and iNOS in astrocytes. ***A***, Staining for HIF-1α in *GFAP-Cre:HIF-1α^fl/fl^* and *HIF-1α^fl/fl^* mice after EAE. ***B***, Quantification of iNOS expression in Mac-2+ macrophages and GFAP-expressing astrocytes at peak EAE in *HIF-1α^fl/fl^* mice (*n* = 5). Area = 0.056 mm^2^. Data are presented as mean ± SEM from *n* = 5 mice. Statistical analysis was performed using an unpaired *t*-test with Welch’s correction (*p* = 0.003*^°^*). ***C***, Quantification iNOS+ cells at peak EAE in *HIF-1α^fl/fl^* (*n* = 5) and *GFAP-Cre:HIF-1α^fl/fl^* (*n* = 4) mice. Area = 0.056 mm^2^. Data are presented as mean ± SEM. (*continued on page 10*). Statistical analysis was performed an unpaired *t* test (*p* = 0.034*^p^*). ***D***, Double-immunofluorescence of GFAP (green) and iNOS (red) in *HIF-1α^fl/fl^* and *GFAP-Cre:HIF-1α^fl/fl^* mice at peak EAE. Statistical analysis was performed using an unpaired *t* test (*p* = 0.01*^q^*). ***E***, Quantification of iNOS+ GFAP+ cells reveals reduced iNOS-expressing astrocytes in *GFAP-Cre:HIF-1α^fl/fl^* (*n* = 4) compared with *HIF-1α^fl/fl^* (*n* = 5) mice. Area = 0.056 mm^2^. Statistical analysis was performed using an unpaired *t* test (*p* = 0.0159*^q^*). ***F***, Quantification of GFAP+ astrocytes shows similar numbers of astrocytes in *GFAP-Cre:HIF-1α^fl/fl^* (*n* = 4) and *HIF-1α^fl/fl^* (*n* = 5) mice at peak EAE. Area = 0.056 mm^2^. Data are presented as mean ± SEM. Statistical analysis was performed using an unpaired *t* test (*p* = 0.06*^r^*). Superscript letters refer to the statistical results in Tables 1, 2, and Results.

**Figure 5:**
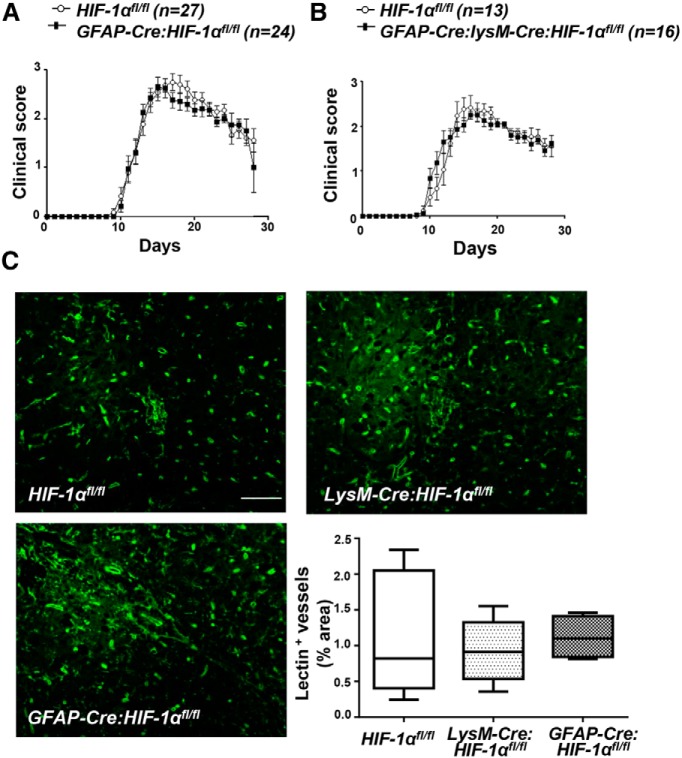
Genetic depletion of HIF-1α in astrocytes or in both astrocytes and myeloid cells does not affect the clinical course of EAE. ***A***, Clinical scores of mice genetically depleted for HIF-1α in astrocytes *GFAP-Cre:HIF-1α^fl/fl^* (*n* = 24) and control mice *HIF-1α^fl/fl^* (*n* = 27). ***B***, Clinical scores of mice genetically depleted for HIF-1α in astrocytes and myeloid cells *GFAP-Cre:lysM-Cre:HIF-1α^fl/fl^* (*n* = 16) and control mice *HIF-1α^fl/fl^* (*n* = 13). ***A*** and ***B***, For each genotype, day of onset, maximum clinical score, and percentage of mice developing signs of paralysis were calculated using mixed linear effects models, and the results are presented in Table 1. Data are presented as mean ± SEM. ***C***, Immunostaining for tomato-lectin shows no significant differences in lectin+ vessels between *HIF-1α^fl/fl^* (*n* = 5), *lysM-Cre:HIF-1α^fl/fl^* (*n* = 5), and *GFAP-Cre:HIF-1α^fl/fl^* (*n* = 4) at peak EAE. Data are expressed as percentage of lectin+ area per mouse and presented as mean ± SEM. Statistical analysis was performed using one-way ANOVA (*p* = 0.83*^y^*). Superscript letter refers to the statistical results in Tables 1, 2, and Results.

Immunohistochemistry for HIF-1α revealed a remarkable decrease of HIF-1α in *GFAP-Cre:HIF-1α^fl/fl^* mice, suggesting that astrocytes are a major source of HIF-1α in the white matter during EAE (Fig. 4A). We further characterized how cell-specific depletion of HIF-1α in astrocytes affected the expression of iNOS, a HIF-1α-target gene. In control *HIF-1α^fl/fl^* mice, iNOS was mainly expressed by astrocytes at the peak of EAE (Fig. 4B). In accordance with reduced expression of HIF-1α (Fig. 4A), the total number of iNOS-expressing cells was decreased in *GFAP-Cre:HIF-1α^fl/fl^* mice compared with *HIF-1α^fl/fl^* control mice (Fig. 4C). At the peak of EAE in *GFAP-Cre:HIF-1α^fl/fl^* mice, iNOS was significantly reduced in astrocytes (Fig. 4D,E), although no differences were observed in the total number of GFAP-expressing astrocytes (Fig. 4F). Overall, these results suggest that astrocyte expression of HIF-1α and its downstream gene target iNOS are reduced in *GFAP-Cre:HIF-1α^fl/fl^* mice. Despite the effect of HIF-a genetic depletion on astrocytic iNOS, no changes were observed in the severity of clinical symptoms of EAE (Fig. 5A; Table 1). Indeed, clinical signs, day of onset, maximum clinical score, and percentage of paralysis were also similar in *GFAP-cre:lysM-Cre:HIF-1α^fl/fl^* and *HIF-1α^fl/fl^* mice (Fig. 5B; Table 1), suggesting that depletion of HIF-1α in both astrocytes and monocytes does not affect neuroinflammatory disease. Although *GFAP-cre:lysM-Cre:HIF-1α^fl/fl^* mice showed decreased percentage of paralysis, the effect was not significant (Table 1). HIF-1α depletion in oligodendrocytes is involved in white matter angiogenesis (Yuen et al., 2014). However, mice with astrocyte or myeloid-specific deletion of HIF-1α had no significant differences in vascular density after EAE (Fig. 5C). Overall, these results suggest that astrocyte and/or myeloid depletion of HIF-1α is not essential for the development of EAE.

## Discussion

In this study, we described that HIF-1α expression is induced in astrocytes and microglia/macrophages in the spinal cord of EAE mice at the peak of the disease. In accordance, transcription of HIF-1α target genes *iNOS* and *EPO*, involved in neuronal damage and protection, is increased. Unexpectedly, we found that genetic depletion of HIF-1α in astrocytes and cells of the myeloid lineage does not affect the development of neuroinflammatory disease. Accordingly, the extent of spinal cord inflammation and demyelination was similar between conditional knock-out and control mice. Together with two recently published studies showing a role for oligodendrocyte-specific HIF-1α in myelination (Yuen et al., 2014) and T-cell-specific HIF-1α in EAE (Dang et al., 2011), our results demonstrate a tightly regulated cellular specificity for HIF-1α contribution in nervous system pathogenesis.

Genetic manipulation of HIF-1α in classical animal models of inflammation and neurodegeneration, including sepsis (Peyssonnaux et al., 2007; Thiel et al., 2007), rheumatoid arthritis and chronic cutaneous inflammation (Cramer et al., 2003) and stroke (Baranova et al., 2007) have shown that HIF-1α exerts a profound influence on disease progression. Therefore, we hypothesized that HIF-1α deficiency in glial cells would likely be protective and HIF-1α overexpression would exacerbate inflammation in the EAE mouse model. However, our EAE experiments conclusively show that genetic ablation of HIF-1α in astroglia and macrophages does not alter disease progression. It is possible that HIF-1α deficiency or exacerbation in astrocytes did not alter EAE progression because of the additional cell sources expressing HIF-1α, including oligodendrocytes and T cells. Indeed, a recent study showed that mice lacking HIF-1α in CD4^+^ T cells are deficient in IL-17 production and are more resistant to EAE (Dang et al., 2011). Furthermore, HIF-1α signaling in oligodendrocytes is essential for postnatal myelination (Yuen et al., 2014). Depending on the cell source, HIF-1α might have positive and negative effects in inflammatory demyelination. On one hand, HIF-1α could exacerbate pathogenesis by increasing BBB opening and inflammatory infiltration. On the other hand, HIF-1α may also have protective effects on oligodendrocyte and neuronal survival. Genetic ablation of HIF-1α might cancel out its diverse effects, thus preventing measurable effects in clinical signs and disease pathogenesis. Last, another possible explanation for the lack of effect of HIF-1α genetic manipulation in EAE could be the partial overlapping functions between HIF-1α and its homolog HIF-2α. Although our study suggests that depletion of HIF-1α in astrocytes or myeloid-lineage cells does not affect vascular density in EAE, depletion of HIF-1α in oligodendrocytes regulates white matter angiogenesis (Yuen et al., 2014). Future studies are required to further characterize the effects of astrocytic and myeloid HIF-1α on inflammatory infiltrate subsets by FACS and angiogenic processes, such as BBB permeability, which can influence CNS inflammation, demyelination, and axonal loss.

Although the use of the human *GFAP-cre* mouse line in our study might have influenced HIF-1α expression in neurons and oligodendrocytes in addition to astrocytes, we did not observe effects on the course of EAE GFAP-cre:*HIF-1α^fl/fl^* mice. Therefore, it is unlikely that potential unspecific and partial reduction of HIF-1α in neurons and oligodendrocytes could have masked the effect of astrocytic HIF-1α depletion. The lack of effect of HIF-1α genetic manipulation in glial cells might stem from the use of the MOG-EAE model used in our study. However, because pharmacological manipulation of HIF-1α influences clinical symptoms in similar models of CNS autoimmunity (Dore-Duffy et al., 2011; Huh et al., 2011), it is likely that alternate HIF-1α-expressing cell types might contribute to inflammatory demyelination. In contrast to the *GFAP-cre* mouse line that may target additional cell types, the use of *lys-cre* line might not fully achieve HIF-1α depletion in the myeloid lineage. Therefore, future studies in recently generated microglial-specific cre mice (Parkhurst et al., 2013) could increase the HIF-1α depletion and shed light in microglial-specific depletion of HIF-1α in inflammatory demyelination.

Our findings that HIF-1α is upregulated in astrocytes and inflammatory cells of white matter areas are in agreement with studies in MS patients. HIF-1α and its downstream genes are upregulated in the brains of MS patients. We observed primarily changes in protein expression of HIF-1α, suggesting that post-translational modifications that stabilize HIF-1α are primarily involved in HIF-1α upregulation in EAE. HIF-1α stabilization may reflect a reactive change within cells consecutive to the hypoxic and inflammatory conditions and might play a causative role in the formation of demyelinating plaques. Indeed, we observed significant increase in HIF-1α levels at the peak of EAE and did not detect HIF-1α upregulation at the onset of the disease. It is possible that HIF-1α accumulation is either too low to be detected at early stages or occurs after the onset of EAE. Future studies using sensitive methods to detect upregulation of HIF-1α at early stage in EAE would be crucial to determine its relationship with early tissue alterations.

In summary, our data demonstrate that deleting HIF-1α in astrocytes and/or myeloid cells or deleting its negative regulator VHL in myeloid cells is insufficient to affect the clinical course of EAE. Further experiments targeting HIF-1α in other glial cell types, such as oligodendrocytes or T-cell subsets, are needed to fully understand the function of HIF-1α in inflammatory demyelinating diseases.

## Synthesis

The decision was a result of the Reviewing Editor Michael Sofroniew and the peer reviewers coming together and discussing their recommendations until a consensus was reached. A fact-based synthesis statement explaining their decision and outlining what is needed to prepare a revision is listed below. The following reviewers agreed to reveal their identity: David Pleasure and Stephen Crocker

### Reviewer #1 concerns:

This manuscript is clearly written and interesting. It demonstrates that genetic manipulation either up or down of HIF1alpha in astrocytes and in monocytes/microglia. There are a number of ways in which this manuscript might be improved:

1) did the authors use a human GFAP promoter-driven cre or a mouse GFAP promoter-driven cre? It is important to know this to be able to judge the specificity of the recombination. Were there evidences of recombination with the GFAP-cre in oligodendroglia, ependyma, or neurons, as well as in astros?
  Based on the Bejenaru 2002 reference provided, it would seem that the GFAP-Cre strain was the small human GFAP promoter that is well known to target many neurons and some oligodendrocytes. The authors need to clearly define what GFAP-Cre line was used and which cell types are targeted based on reporter gene expression studies. They then also need to discuss the implications of targeting Cre-recombinase activity to cells other than astrocytes for the interpretation of their findings. Since much of the Cre effects were negative, the aberant targeting may not be of substantial consequence, but it needs to be acknowledged and discussed.
2)it is interesting that manipulating HIF1alpha in monocytes (and microglia) failed to alter MOG peptide EAE. Since monocytes are major effectors of EAE damage, and HIF1alpha is involved in their behavior, it would be important to characterize the inflammatory infiltrates in the lysMcre recombined vs control mice in greater detail than simply CD3 immunostaining, either by immunostaining or preferably by FACS.
3) is it valid to use two constitutively expressed cre transgenes simultaneously, as the authors mention doing? Or is it possible that artifacts are induced thereby?
4)Figure 2A is said to show neuronal HIF1alpha immunostaining, but does not clearly show it. In Figure 2C, why are the patterns of HIF1alpha immunostaining different in the fields co-immunostained for monos/microglia and for astroglia?
5) how was hypoxia induced in the hypoxic brain control?
6) Yuen et al (2014) reported marked white matter angiogenesis in mice in which HIF1alpha was stabilized in the oligodendroglial lineage. Were there changes in white matter blood vessels in the GFAPcre and/or LysMcre experiments?

### Reviewer #2 concerns:

The authors provide an interesting set of data to indicate that astrocytic or myeloid HIF1a expression contributes minimally to the protection of the CNS in a model of inflammatory demyelination. The study was well written and the hypothesis and procedures clearly presented.

The strengths of this study lie in the clinical comparisons of EAE treatment groups and the accompanying pathology.

There were two weakness of this study.

(1) First was the absence of any primary culture validation of the transgenic knockouts which would validate the deletion in the specific cell types.
(2) Second, the depth of analysis on the impact of cell-specific deletion of HIF1a could have been bolstered by an analysis of a target gene. Simply stated, there were no analyses of HIF1a target genes (as shown in Fig 1) in the conditional knockouts to suggest what the contribution of astrocytic or myeloid cells toward iNOS expression or EPO during EAE. Inclusion of these data would aid in future interpretations by providing a new perspective on how the cellular source of specific products (such as iNOS) may critical to interpreting measurable global changes that can occur during disease. These data may further increase the potential impact of this study.
